# Subordination of lived expertise: A new policy threat to federally funded mental health research

**DOI:** 10.1371/journal.pmen.0000661

**Published:** 2026-07-16

**Authors:** Ernesto Isaac Lara

**Affiliations:** Independent, Boston, United States of America; PLOS: Public Library of Science, UNITED KINGDOM OF GREAT BRITAIN AND NORTHERN IRELAND

## Introduction

Mental health research is fundamentally political. Hegemonic institutions based in the Global Minority (i.e., the “Global North” holding disproportionate power despite demographic minority status) have long claimed authority to define scientific rigor, evidence, and epistemic legitimacy, erasing the lived realities and knowledge system built by the Global Majority. The White House Office of Management and Budget’s (OMB) recent 412-page proposal intensifies this epistemic injustice by positioning political appointees as the ultimate authority on all federally funded research [1,2].

Under the guise of “transparency and accountability,” the proposal would fundamentally restructure federal grant management across key mental health research funding agencies, including the National Institutes of Health, National Science Foundation, and Department of Health and Human Services. Mechanistically, it would effectively gatekeep whose research can be published and disseminated at scale by reducing peer-review to “advisory status;” shifting the financial burden of open-access publishing to individual researchers and institutions; and further restricting international research collaboration. Most concerningly, it explicitly targets research on diversity, equity, inclusion (DEI), and other work deemed “not aligned with the President’s agenda.”

This act of what many view as political censorship builds upon a broader assault on knowledge production. A recent series of executive orders have essentially banned DEI research, restricted work on our most vulnerable communities, and mandated all federally research align with governing political agendas [3]. The Administration’s attempted $2 billion cut in funding for the Substance Abuse and Mental Health Services Administration (SAMHSA) signals a deliberate pattern of defunding research and programs dedicated to combating health inequities and structural barriers of care [4,5].

Psychiatricization and sanism are mechanisms by which mental health research and services weaponize mental health, creating labels for illness and pathologizing resistance as a means of control and oppression over marginalized communities [6,7]. Across contexts, psychiatric labels have served as a means of oppression, from diagnosing enslaved people with “drapetomania” for seeking freedom, to contemporary forced treatment and institutionalization as a tool of policing [8,9]. The OMB’s proposal formalizes “the system working according to design,” no longer hiding under proclaimed neutrality, but codifying epistemic control explicitly as policy. For mental health research centering lived experience, health equity, and systems change, this proposal represents an existential threat.

## How the OMB proposal formalizes state control of mental health research

The proposal systematically targets research that challenges American hegemony and its role as a driver of oppression. Research on topics that critically questions psychiatry, sanism, biomedicine, and other structural barriers to mental wellbeing would be especially vulnerable, potentially being dismissed as “purely activism” rather than recognized as legitimate scholarship.

This attack operates through a cascade of gatekeeping mechanisms. Political appointees effectively gain veto power over research agendas and funding, meaning lived experience scholarship would become vulnerable to ideological rejection before methodological evaluation. Already, within the mental health research space, researchers who combine their technical and lived expertise face heightened scrutiny, often having their scientific knowledge routinely mischaracterized as “activism” [10–12].

Enhanced publication gatekeeping further compounds this harm. Academic publishing has historically been inaccessible, especially for researchers from the Global Majority facing structural barriers [13]. By eliminating federal funding for open-access publication fees, the proposal strips a critical funding stream for Global Majority researchers and community-based mental health organizations, shifting costs to institutions already operating with minimal resources.

International collaboration restrictions will further isolate Global Majority researchers from US networks and funding. The Administration’s previous cuts to international research funding, including the decimation of the United States Agency for International Development (USAID) and defunding of global mental health initiatives, have already fragmented these networks [14]. Communities that have built lived experience infrastructures and invested in community-based forms of care risk losing access to the resources that US collaboration provided. The OMB proposal exacerbates this vulnerability by enabling political appointees to terminate funding any time without cause, on a political whim. For lived experience communities building collective knowledge and mutual aid networks, this legislation would destabilize research partnerships, erode trust, and deepen isolation.

Rather than codify censorship, policy reform can and must support the work of the lived experience community by reimagining federal research infrastructure itself. Within academic publishing, this would mean revising the peer review and publication mechanisms that already function as gatekeepers serving the Global Minority’s interests [13]. Within grants and funding, it would manifest as protected and dedicated funding streams for lived experience led research initiatives. Ultimately, it would center community control of the research agenda, ensuring that lived experience foundationally shapes and drives ethical, accountable science [6,15]

## In defense of our expertise: A call to action

The OMB is accepting public comments through July 13, 2026 at regulations.gov/document/OMB-2026-0034-0001, providing us an opportunity to advocate for the research our communities need [1].

Beyond submitting an individual comment, we can take further action by collectively organizing within our networks. When submitting a comment, individually and/or collectively, ground it in your and your communities’ lived expertise: How would this proposal harm your communities? What knowledge are they creating and how would it become unfundable and unpublishable? Throughout your messaging, make it clear that lived expertise is rigorous science, not a “nice to have” nor “just advocacy.”

Your voice, your lived expertise matters. Submit a comment by July 13th and mobilize your networks to do the same.
